# Brain structure changes associated with sexual orientation

**DOI:** 10.1038/s41598-021-84496-z

**Published:** 2021-03-03

**Authors:** Mikhail Votinov, Katharina S. Goerlich, Andrei A. Puiu, Elke Smith, Thomas Nickl-Jockschat, Birgit Derntl, Ute Habel

**Affiliations:** 1 Institute of Neuroscience and Medicine 10, Research Centre Jülich, Jülich, Germany; 2 Department of Psychiatry, Psychotherapy and Psychosomatics, Medical Faculty, RWTH Aachen University, Aachen, Germany; 3 Department of Biomedical Sciences of Cells and Systems, Section Cognitive Neuroscience, University Medical Center Groningen, University of Groningen, Groningen, The Netherlands; 4 Department of Psychology, Biological Psychology, University of Cologne, Cologne, Germany; 5 Department of Psychiatry, Iowa Neuroscience Institute, University of Iowa, Carver College of Medicine, Iowa City, IA USA; 6 Department of Neuroscience and Pharmacology, Carver College of Medicine, University of Iowa, Iowa City, IA USA; 7 Department of Psychiatry and Psychotherapy, Medical School, University of Tübingen, Tübingen, Germany

**Keywords:** Neuroscience, Sexual behaviour, Sexual dimorphism

## Abstract

Biological sex differences in brain function and structure are reliably associated with several cortico-subcortical brain regions. While sexual orientation (hetero- versus homosexuality) has been similarly linked to functional differences in several phylogenetically-old brain areas, the research on morphological brain phenotypes associated with sexual orientation is far from conclusive. We examined potential cerebral structural differences linked to sexual orientation in a group of 74 participants, including 37 men (21 homosexual) and 37 women (19 homosexual) using voxel-based morphometry (VBM). Gray matter volumes (GMV) were compared with respect to sexual orientation and biological sex across the entire sample using full factorial designs controlling for total intracranial volume, age, handedness, and education. We observed a significant effect of sexual orientation for the thalamus and precentral gyrus, with more GMV in heterosexual versus homosexual individuals, and for the putamen, with more GMV in homosexual + than heterosexual individuals. We found significant interactions between biological sex and sexual orientation, indicating that the significant effect for the putamen cluster was driven by homosexual women, whereas heterosexual women had increased precentral gyrus GMV. Heterosexual men exhibited more GMV in the thalamus than homosexual men. This study shows that sexual orientation is reflected in brain structure characteristics and that these differ between the sexes. The results emphasize the need to include or control for potential effects of participants’ sexual orientation in neuroimaging studies. Furthermore, our findings provide important new insights into the brain morphology underlying sexual orientation and likely have important implications for understanding brain functions and behavior.

## Introduction

In the last couple of decades, there has been intense debate over sex and gender and over how societies perceive and categorize sexual roles and sexual behaviors. Developments in neuroimaging methods allowed us to push research on this topic further and investigate the functional and structural neural phenotype underlying gender and sexual behavior and, in particular, sexual orientation.

Sex differences in brain morphology have been extensively documented in neurocognitive and behavioral domains^1,2^. Differences are reported in total brain volume, gray and white matter proportions and volumes, as well as in tissue densities of several phylogenetically old structures including the insula, hippocampus, thalamus, putamen, amygdala, and the cerebellum^3–7^. While these findings may imply a greater probability of innate encoding of reproductive and self-preservation behaviors (including sexual orientation), we cannot rule out the contribution of plasticity in shaping sexual interests and orientation in that, for instance, steroid hormone profiles can shape the development of male versus female neural phenotypes^8^. Morphological and functional bases of sexual behavior, however, are scarcely investigated.

Reproductive behaviors (i.e., sexual or parental behavior) along with self-preservation behaviors (i.e., eating and drinking) are intrinsically rewarding and encoded in phylogenetically old subcortical structures including the thalamus, hypothalamus, amygdala and the striatum, with each region playing specific roles, such as reward expectation and evaluation, affective processing, guiding to foraging, etc. (for an overview, see^9^). Evidence for the involvement of these regions comes from studies where already in the phylogenetically oldest group of vertebrates (i.e., cyclostomes) it was observed that basic components of the basal ganglia in lampray are similar to those of mammals, and that the circuit features, molecular markers, and physiological activity patterns were conserved^10,11^. Moreover, this system is involved in behaviors like value-based decisions^11^, reward, appetitive-seeking or avoidance behaviors^12^ across species (see review^13^,). Contrastingly, secondary rewards (i.e., monetary incentives) are represented in ontogenetically newer brain regions, like the prefrontal cortex^14^. Meta-analytic findings confirmed the involvement of these structures in sexual behaviors^15^. The same regions have been identified when investigating the association between sexual orientation and brain morphology and function. For instance, early studies reported an almost two-fold volumetric increase of the suprachiasmatic nucleus of the hypothalamus^16^ but twice smaller volumes of the third interstitial nucleus of the anterior hypothalamus (INAH-3) in homosexual (HoM) relative to heterosexual men (HeM) and no volumetric differences in the INAH-3 between heterosexual women (HeW) and HoM^17^. Moreover, the size of the midsagittal plane of the anterior commissure has been associated with both biological sex and sexual orientation^18^. Although these findings are not equivocally replicated and do not highlight causal relationship between morphological differences and sexual orientation or vice-versa, they do strengthen the notion that, much like sex differences in brain structure and function, sexual orientation is strongly biologically anchored. They also stress the need for further studies investigating midline brain structures in relation to biological sex and sexual orientation^19^.

Comparing the regional cortical thickness and subcortical volumes of heterosexual and homosexual males revealed smaller thalamus volumes and thinner right orbitofrontal and right visual cortices in HoM^20^. No significant morphological differences were found between HeW and HoW in these areas. The same regions (in particular the lingual and pericalcarine gyrus and cuneus), however, were thinner in HeW relative to HeM. Similarly, in a multimodal MRI study, HoM displayed thicker anterior cingulate cortex, precuneus, and left occipito-termporal cortex relative to HeM and HeW^21^. Furthermore, HoM displayed stronger cortico-subcortical covariations between these regions and stronger functional connectivity between the thalamus and hypothalamus in comparison to heterosexual men. While arguably HoM may show more “female characteristics” with respect to showing similar cortical thickness to HeW, these features differ from those of HeM. In another voxel-based morphometry (VBM) study on sexual orientation, less gray matter density was observed in the ventral cerebellum, the left ventral premotor cortex, and the temporo-basal cortex in homosexual relative to heterosexual women^22^. While no morphological differences were reported between HoM and HeM, the left medial temporal lobe showed reduced grey matter in HeM than in HeW, suggesting this area has a more “male-like” structural pattern in HoW. In addition to local volume differences, sex-atypical cerebral asymmetry and functional connections were observed in HoM compared to heterosexual men and women, indicating a rightward cerebral asymmetry in HeM and HoW but symmetrical volumes of the hemispheres in HoM and HeW^23^.

Although these findings show broad functional and morphological differences in cerebral areas between HoM and HeM, some structural similarities appear consistent between HoM and HeW. These findings are supported by neurocognitive data showing less asymmetry and a relative feminization of fluid intelligence in HoM compared to HeM^24^. Similarly, a meta-analysis found a cross-sex-shift in cognitive performance in that HoM perform similar to HeW, while HoW perform similar to HeM^25^. Furthermore, homosexuality seems to be associated with less distinct cerebral sexual differentiation^26^.

Taken together, although extant findings suggest that human sexual orientation is associated with brain morphology, the heterogeneous and limited number of studies precludes a thorough understanding of the shared and distinct neural signatures of sexual orientation in men and women. Here, we aimed to analyze sex-specific differences in regional gray matter volumes associated with sexual orientation. Based on previous findings of morphological differences in subcortical limbic and sensorimotor areas, particularly the thalamus and hypothalamus, we hypothesized that gray matter volumes in these areas would be associated with sexual orientation. Specifically, we predicted that (1) the thalamus and cingulate gyrus will show volumetric differences between HeM and HoM, and (2) the premotor and temporo-basal cortices will show volumetric differences when comparing HeW and HoW.

## Methods

### Participants

MRI data of 37 men (21 homosexual men = HoM; 16 heterosexual men = HeM) and 37 women (19 homosexual women = HoW; 18 heterosexual women = HeW) entered the analyses (see Supplementary Table 1 for sample demographics; N = 74). Participants were recruited through university bulletin boards, in Lesbian, Gay, Bisexual, Transgender, and Queer or Questioning + (LGBTQ +) organizations in Aachen, Cologne and surrounding areas, and by word-of-mouth recommendation. Biological sex was recorded at birth and sexual orientation was assessed by self-report. All participants were asked to indicate their sexual orientation at the time of measurement using a 1 to 4 scale ranging from (1) exclusively homosexual, (2) predominantly homosexual, (3) bisexual, or (4) heterosexual. We only included heterosexual participants who self-identified as exclusively heterosexual. Homosexual participants were included when self-identifying as exclusively and/or predominantly homosexual. Bisexual participants were excluded. Altogether, 19 homosexual men and 15 homosexual women identified as exclusively homosexual, while two homosexual men and four homosexual women identified as predominantly homosexual. In total, 21 homosexual men and 19 homosexual women took part in the study. Of the 74 participants, five were left-handed (3.7%; two HoW, two HeM, one HeW). Exclusion criteria were axis I disorders as assessed with the fourth edition of the German version of the Diagnostic and Statistical Manual of Mental Disorders (SCID, Wittchen et al. 1997). Further exclusion criteria were conventional MRI exclusion criteria and medical conditions affecting the cerebral metabolism. The study was approved by the local Ethics Committee of the Medical Faculty of the RWTH Aachen University (EK 088/09) and were in accordance with the Helsinki declaration (1964) and its later amendments or comparable ethical standards. All participants gave informed consent prior to participation and received financial compensation for their participation in the study.

### Analysis of behavioral data

We tested the normality of the questionnaire data using Shapiro Wilk tests. Multivariate analyses of covariance (MANCOVAs) were performed to compare each questionnaire between the four groups, with the respective questionnaire scores as dependent variables, and sexual orientation and biological sex as fixed factors. Each MANCOVA contained age, handedness and educational level as covariates of no interest to adjust for potential effects of these variables.

### Questionnaires

The Bem Sex Role Inventory (BSRI), the German version of the Inventory of Clinical Personality Accentuations (IKP;^27^ the NEO Five-Factor Inventory (NEO-FFI^28^,) and Toronto Alexithymia Scale (TAS-20^29^, were assessed in each participant. These data were collected within the scope of a larger study^19,30,31^. The Bem Sex Role Inventory (BSRI) was used to measure gender expression and roles in terms of masculinity and femininity^32^. The BSRI is a self-report scale that assesses 60 personality traits using a 7-point Likert scale, 20 of which are thought to reflect masculinity, 20 femininity, and 20 gender neutrality. The scale has good psychometric properties with alpha coefficients of 0.78 for the femininity sub-scale, and 0.87 for the masculinity sub-scale as well as a high test–retest reliability^33^. The German version of the Inventory of Clinical Personality Accentuations (IKP) was administered for a clinical personality characterization of the sample^27^. The IKP comprises 132 items derived from the DSM-IV and the ICD-10 that assess 11 personality accentuations dimensionally (paranoid, dependent, impulsive-explosive, schizoid, narcissistic, borderline, avoidant, compulsive, schizotypal, antisocial, and histrionic dimension). The NEO Five-Factor Inventory (NEO-FFI) was used to assess the ‘Big Five’ personality traits (neuroticism, extraversion, openness, agreeableness, and conscientiousness)^28^. In addition, levels of the personality dimension alexithymia were assessed using the 20-item Toronto Alexithymia Scale (TAS-20) which measures the degree to which a person has difficulties identifying their feelings, describing them to others, and exhibiting an externally oriented cognitive style^29^. Supplementary Table 2 shows the means and standard deviations for each of the domains outlined above (BSRI, ICP, NEO-FFI, and TAS-20) for all groups.

### Structural imaging data

#### MR procedure and preprocessing

Structural magnetic resonance imaging data were obtained using a 3-T PRISMA MR scanner (Siemens Medical Systems, Erlangen, Germany) located in the Department of Psychiatry, Psychotherapy and Psychosomatics, RWTH Aachen University Hospital. T1-weighted structural images were acquired with a 20-channel head coil by means of a three-dimensional magnetization-prepared rapid acquisition gradient echo image (MPRAGE) sequence (voxel size: 1 × 1 × 1 mm, 256 × 256 matrix, FoV: 256 × 256 mm 176 slices, TR = 1900 ms, TE = 2.52 ms, flip angle = 9).

Imaging data were preprocessed using the Computational Anatomy Toolbox (CAT12) implemented in SPM12 (Wellcome Department of Imaging Neuroscience Group, London, UK; http://www.fil.ion.ucl.ac.uk/spm) and running under MATLAB 2012b (The Math-Works, Natick, MA). We used the default settings of CAT12 as the toolbox shows improved sensitivity for detecting differences in grey matter volume (GMV) compared to VBM8^34^. 3D T1-weighted MRI scans were reoriented to the intercommissural plane, corrected for field intensity inhomogeneities, and spatially normalized onto MNI standard space. Diffeomorphic Anatomical Registration Through Exponentiated Linear Algebra Algorithm (DARTEL) was used for normalization^35^ because DARTEL provides more precise spatial normalization to the template than the conventional algorithm^36^. Images were then segmented into gray matter (GM), white matter (WM), and cerebrospinal fluid (CSF), and modulated with Jacobian determinants. Finally, the modulated GM volumes were smoothed with a Gaussian kernel of 8 mm full width at half maximum (FWHM). A homogeneity check identified no outliers, thus the GM volumes of all 74 participants (21 HoM, 16 HeM, 19 HoW, 18 HeW) were included in subsequent analyses.

### VBM analyses: sexual orientation

First, whole-brain analyses were performed using full factorial designs to compare GMV with respect to sexual orientation (whole sample and per biological sex, i.e. women vs. men), including age, total intracranial volume (TIV), education, and handedness as regressors of no interest. Additional conjunction analyses were performed to identify overlaps and differences in GMV with respect to sexual orientation in the whole sample and broken down by biological sex. For all analyses, we applied Family-Wise Error (FWE) corrections at the voxel level (*p* < 0.05) to correct for multiple comparisons. Coordinates in tables represent local maxima for each cluster or subcluster.

### Connectivity modelling and functional decoding

The functional properties of a given brain region largely rely upon its connectivity profile. To detect the functional fingerprint of the regions identified by our VBM analyses, we investigated functional connectivity patterns irrespective of task or rest. To this end, we used task-dependent (meta-analytic connectivity modeling, MACM) and task-independent (resting-state fMRI) connectivity analyses for each of the regions retrieved (please refer to^37–39^ for further details). Functional fingerprints were identified using the behavioral domain and paradigm class meta-data categories from the BrainMap database^38,40^; http://www.brainmap.org). These describe classes of mental processes isolated by the statistical contrasts computed in the experiments analyzed. Behavioral domains denote mental processes isolated by the respective contrast, whereas behavioral paradigm classes categorize the tasks employed in the specific studies.

Additionally, we conducted two further exploratory analyses:

### Overlay with the NeuroQuery-derived model

NeuroQuery is a recent online automated meta-analytical tool based on supervised machine learning techniques fitted over 13,000 full-text publications. The tool assembles results from the literature into a brain map using free-text queries or single terms. The ensuing outputs are predictions of the likelihood of observed brain locations (rescaled by their standard deviation)^41^ that are less sensitive to terminology variations. NeuroQuery thus extends the scope of our MACM analyses by extracting from the literature a comprehensive statistical summary of evidence accumulated by neuroimaging research. Since the tool was published after we completed data collection, we did not use NeuroQuery’s results output to the “sexual orientation” query as a predictor. Instead, we overlaid our VBM findings onto the map obtained from NeuroQuery based on the term “sexual orientation” (https://neuroquery.org/query?text=%22sexual+orientation%22) yielding 40 studies to examine if our results hold against functional and structural papers related to the term.

### ROI analyses: relationship between extracted GMV from clusters and gender roles self-concepts

In subsequent exploratory analyses, we investigated whether the identified GMV clusters were significantly related to the participants’ self-concept of gender roles. VBM ROI gray matter analyses were performed on clusters emerging from the heterosexual > homosexual and the reverse contrast and masculinity and femininity scores from the BSRI questionnaire. Mean parameter estimates were extracted using MarsBaR (http://marsbar.sourceforge.net/) and entered into SPSS 20 analyses (SPSS, Chicago, IL). Partial one-tailed correlations were performed between GMVs and gender identity scores (masculinity, femininity) with age, TIV and handedness as covariates of no interest.

## Results

The four groups (HoM, HeM, HoW, and HeW) did not differ in age (age range 19 to 54 years) or educational level (*p* > 0.05 for all tests; see Supplementary Table 1).

### Personality data

MANCOVA analyses for BSRI masculinity and femininity scores showed a significant main effect of biological sex (V_(Pillai’s trace)_ = 0.11, F(2, 64) = 3.95, *p* = 0.024) and a significant interaction between biological sex and sexual orientation (V_(Pillai’s trace)_ = 0.11, F(2, 64) = 3.99, *p* = 0.023). Moreover, there was a significant main effect of biological sex on masculinity (F(1,64) = 8.08, *p* = 0.006), confirming an overall more masculine self-concept of gender identity in men than in women (men = 4.96 ± 0.09; women = 4.62 ± 0.09). HeM had an overall more masculine self-concept of gender identity than HoM (*p* = 0.006) and HeW (*p* < 0.001); see supplementary Table 2. In addition, masculinity scores were negatively associated with age (V_(Pillai’s trace)_ = 0.118, F(1,64) = 6.66, *p* = 0.012), indicating that men’s self-concept of masculinity decreased with age. For the BSRI femininity scores, no significant effects were observed between the groups *(p* > 0.05 for all tests)*.*

For clinical personality accentuations (IKP), MANCOVA analyses yielded a significant main effect of biological sex (V_(Pillai’s trace)_ = 0.364, F(11,51) = 2.65, *p* = 0.009) on narcissistic (*p* = 0.021), schizotypal (*p* = 0.025), and antisocial personality accentuations (*p* = 0.003), suggesting that men were overall more narcissistic, schizotypal, and antisocial than women. No main effects of sexual orientation were observed (all *p* > 0.05). However, there were significant interactions between biological sex and sexual orientation regarding avoidant (*p* = 0.005), schizoid (*p* = 0.029), and compulsive (*p* = 0.043) personality accentuations. Follow-up pairwise comparisons revealed that sexual orientation had significant opposite effects on avoidant personality accentuation, depending on biological sex: HoM were significantly more avoidant than HoW (HoM mean = 53.33, HoW mean = 43.33; *p* = 0.002), whereas this difference was not significant for HeM compared to HeW (HeM mean = 45.36, HeW mean = 43.33; *p* = 0.065). The analyses of the TAS-20 alexithymia scores revealed a main effect of sexual orientation on TAS-20 total scores (V_(Pillai’s trace)_ = 0.27, F(4,57) = 5.34, *p* < 0.001), which was solely driven by the TAS-20 subscale externally oriented thinking (EOT; *p* < 0.001). These results suggest that HoM and HoW have a significantly more externally oriented thinking style than HeM and HeW, resulting in overall higher alexithymia levels for homosexual vs. heterosexual individuals. Biological sex was not associated with the alexithymia scores and there was no significant interaction between biological sex and sexual orientation (*p* > 0.05).

MANCOVA analyses on the NEO-FFI scores showed a significant interaction between biological sex and sexual orientation (V_(Pillai’s trace)_ = 0.22, F(5,50) = 0.28, *p* = 0.024). The main effect of sexual orientation did not reach significance (*p* = 0.085), and there was no effect of biological sex (*p* = 0.159). The interaction between biological sex and sexual orientation was significant only for neuroticism (*p* = 0.002) with HeW scoring higher on neuroticism than HoW (*p* = 0.005), whereas HoM and HeM did not differ in neuroticism (*p* > 0.1; see supplementary Table 2 for details).

### VBM results

#### Whole sample

**Figure 1:**
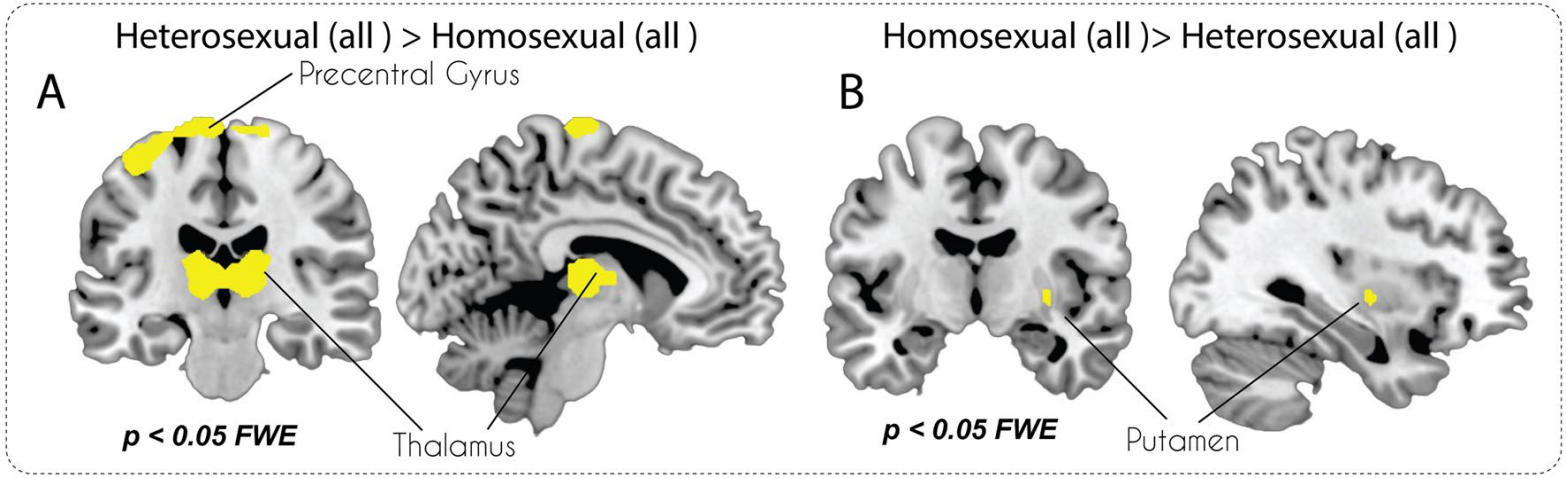
Whole-brain v-FWE corrected VBM analyses for the entire sample.

Whole-brain analyses (thresholded at *p* < 0.05 FWE-corrected at voxel level) revealed increased GMV in the thalamus, postcentral gyrus, middle occipital gyrus, precentral gyrus, middle temporal gyrus, and the cerebellum in heterosexual compared to homosexual participants (see Fig. 1A and Table 1). The reverse contrast revealed one significant cluster in the putamen (see Fig. 1B, Table 2).

**Table 1:** Cluster list for contrast heterosexual > homosexual all group (threshold p < 0.05 FWE corrected at voxel level).

Brain areas (aal)	Side	Cluster size	x	y	z	T
Precentral Gyrus	L	2438	− 41	− 18	66	8,18
SFG	L	s.c	− 5	− 18	80	7,59
Precentral Gyrus	R	s.c	14	− 24	78	7,34
Thalamus	R	2766	9	− 21	9	7,36
Thalamus	L	s.c	− 12	− 20	2	6,61
Thalamus	R	s.c	15	− 18	18	6,51
Postcentral Gyrus	R	147	32	− 30	71	6,50
Middle Occipital Gyrus	R	48	20	− 102	9	6,02
Precentral Gyrus	R	86	57	− 6	45	5,92
Cerebelum	R	136	20	− 81	− 53	5,06
Postcentral Gyrus	R	4	23	− 36	77	4,73
MTG	L	3	− 56	− 69	18	4,66

*L/R* left/right in the brain; *s.c.* sub-cluster, *SFG* Superior Frontal Gyrus; *MTG* Middle Temporal Gyrus.

**Table 2:** Cluster list for contrast homosexual > heterosexual all group (threshold p < 0.05 FWE corrected at voxel level).

Brain areas (aal)	Side	Cluster size	x	y	z	T
Putamen	R	53	32	− 8	2	5,58

*L/R* left/right in the brain.

#### Sex-specific analyses

**Figure 2:**
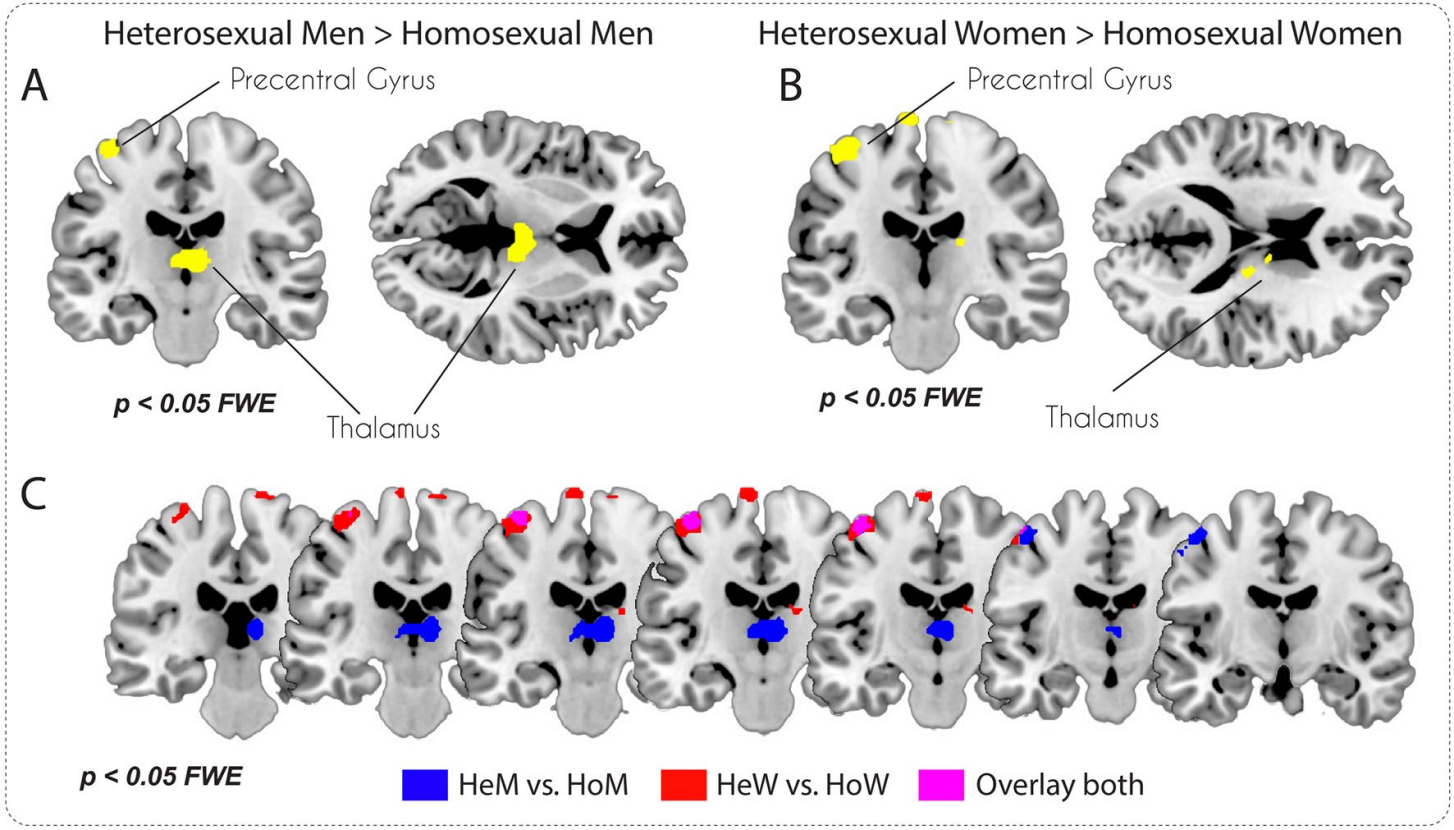
Whole-brain v-FWE corrected sex-specific VBM analyses. (**A**) HeM vs. HoM contrast; (**B**) HeW vs. HoW contrast; (**C**) Overlay of both (**A** and **B**) contrasts. *HeM* heterosexual men; *HoM* homosexual men; *HeW* heterosexual women; *HoW* homosexual women.

The sex-specific comparison between HeM versus HoM and HeW versus HoW revealed the same clusters as the whole sample analysis. The cluster size, however, was larger in the thalamus for men (see Fig. 2A, Table 3), and in the pre- and postcentral gyrus, superior frontal gyrus and caudate nucleus for women (see Fig. 2B, Table 4). Overlaying the contrasts showed an overlap in the precentral gyrus (see Fig. 2C). Moreover, the comparison between HoW and HeW yielded a cluster in the putamen (see Table 5), while the equivalent contrast in men yielded no significant clusters.

**Table 3:** Cluster list for contrast heterosexual males > homosexual males (threshold *p* < 0.05 FWE corrected at voxel level).

Brain areas (aal)	Side	Cluster size	x	y	z	T
Thalamus	R	632	6	− 21	8	6,19
Precentral Gyrus	L	327	− 59	− 5	41	5,79
Precentral Gyrus	L	s.c	− 45	− 12	57	5,79
Precentral Gyrus	L	s.c	− 39	− 20	68	5,74
SFG	L	16	− 5	− 18	80	5,38
MTG	L	3	− 60	− 66	− 6	4,68

*L/R* left/right in the brain; *s.c.* sub-cluster, *SFG* Superior Frontal Gyrus; *MTG* Middle Temporal Gyrus.

**Table 4:** Cluster list for contrast heterosexual females > homosexual females (threshold *p* < 0.05 FWE corrected at voxel level).

Brain areas (aal)	Side	Cluster size	x	y	z	T
Precentral Gyrus	L	488	− 44	− 17	63	6,48
Precentral Gyrus	L	s.c	− 51	− 12	53	5,26
Precentral Gyrus	R	76	14	− 24	78	6,18
SFG	L	142	− 8	− 18	78	5,79
Thalamus	R	94	17	− 20	18	5,53
Caudate Body	R	s.c	9	− 5	17	5,45
Precentral Gyrus	R	24	30	− 29	72	5,28
Postcentral Gyrus	R	15	21	− 33	77	5,22
Precentral Gyrus	L	1	− 33	− 6	68	5,13

*L/R* left/right in the brain; *s.c.* sub-cluster, *SFG* Superior Frontal Gyrus.

**Table 5:** Cluster list for contrast homosexual females > heterosexual females (threshold p < 0.05 FWE corrected at voxel level).

Brain areas (aal)	Side	Cluster size	x	y	z	T
Putamen	R	104	33	− 9	2	5,58

*L/R* left/right in the brain.

### Functional decoding

We performed functional decoding analyses with the clusters emerging from the analyses above as seed regions. Behavioral domains and paradigms that were significantly over-represented among experiments in BrainMap (*p* < 0.05, FDR-corrected for multiple comparisons) were identified for each cluster for hetero- versus homosexual individuals across the whole sample (irrespective of biological sex) and in men and women separately (sex-specific).

#### Cluster 1: Thalamus (MNI 9, − 21, 9)

The heterosexual > homosexual orientation analysis (irrespective of biological sex; whole sample) related this thalamus seed to action execution, perception, somesthesis, pain, and to finger tapping, pain monitoring and discrimination paradigms. In HeM vs. HoM, the thalamus seed was related to action execution, perception, and somesthesis, pain, and to functional paradigms including overt recitation/repetition and reward processing. Last, the HeW vs. HoM analysis showed that the thalamus is functionally associated with broader functional domains including perception, vision, motion, attention, and memory, and to functional paradigms on visual pursuit/tracking, delayed match to sample, and visuospatial attention.

#### Cluster 2: Pre- and postcentral gyrus (MNI − 41, − 18, 66)

Heterosexuality > homosexuality (whole-sample) analyses related the pre-/post-central gyrus to action execution, speech, perception, and somesthesis functional domains, and to paradigms including finger tapping, drawing, flexion/extension, and reward. In HeM vs HoM, the pre-/post-central gyrus was functionally related to action execution, motor learning, perception, and somesthesis, and to paradigms including finger tapping, drawing, and flexion/extension. Last, the HeW vs HoW analysis showed that the pre-/post-central gyrus is functionally associated to action execution, motor learning, perception, and somesthesis, and to paradigms including drawing, grasping, finger tapping, and flexion/extension.

### Overlay with model derived for NeuroQuery

Using the term “sexual orientation” as input to NeuroQuery, we obtained a modelled brain map from 40 matching studies that partially overlapped with our findings from the whole-sample analysis (all heterosexual vs. all homosexual). Specifically, we found significant overlap in the thalamus and precentral gyrus (See supplementary Figure S1).

### ROI analyses: relationship between extracted GMV from clusters and gender roles self-concepts

Partial correlations were performed between GMVs and gender identity scores (masculinity, femininity) with age, TIV and handedness as covariates of no interest. See supplementary Figs. 2, 3 and 4.

## Discussion

In this study we investigated cortico-subcortical gray matter volume differences in homosexual and heterosexual individuals to address the sparse and heterogeneous findings on morphological brain differences associated with sexual orientation. In line with our hypotheses, we found that, relative to homosexual participants, heterosexual individuals had significantly higher GMV in the thalamus and precentral gyrus. Homosexual participants showed higher GMV in the putamen. Breaking these results down by biological sex, highlighted that hetero- versus homosexual women (HeW > HoW) showed larger GMV in the precentral gyrus, while the reverse contrast (HoW > HeW) revealed larger GMV in the putamen. Moreover, homo- versus heterosexual men (HoM > HeM) showed higher GMV in the thalamus. Functional decoding analyses suggested that thalamus and pre-/post-central gyri are involved in broad networks associated with action execution, perception, and somesthesis that spanned somatosensory and higher-order cognitive functions. In addition, HeM had an overall more masculine self-concept of gender identity than all other groups, and HoM and HoW have a significantly more externally oriented thinking style than HeM and HeW. Our results show that sexual orientation has unique effects on brain morphology and that these effects are contingent on biological sex.

Confirming our hypothesis, GMV analyses revealed an association between sexual orientation and GMV in sensorimotor regions. These results are consistent with previous findings in that HoM exhibited smaller thalamus volumes compared to HeM^20^ and that HoW showed smaller thalamic GMV than HeW. Similarly, homosexual individuals exhibited lower GMV in the cerebellum and premotor cortex, irrespective of their biological sex^22^. However, our results did not reveal volumetric differences in orbitofrontal and right visual cortices^20^. Cross-species research emphasized the unequivocal role of the thalamus in complex information processing, exchanging and relaying sensory, motor, and cognitive information to higher-order association areas, integrating information across networks and modulating cortical laminar synaptic activity^42–44^. In heterosexual and homosexual individuals, the mediodorsal thalamus was more activated by faces of the preferred sex relative to faces of a less desired sex, independent of the observers’ biological sex or sexual orientation^45^. While congruently suggesting that hypothalamic activation has downstream effects on visual perception likely impacting the selection of sexual partners^46^, previous findings also support a role of the thalamus in signaling sexual reward^47^ and arousal among homosexual and heterosexual individuals^48^.

The thalamus has numerous reciprocal cortico-thalamic connections with reward and sensory-motor regions^49^. Meta-analytic findings show that the thalamus encodes primary and secondary rewards, reward processing, and reward anticipation^14,50^. Additionally, Poeppl with colleagues highlighted the role of the thalamus in encoding human sexual preference^15^. As such, we believe that the rewarding psychophysical properties of sexual stimuli (i.e., valence, intensity, magnitude, pleasantness,) may likely differ between heterosexual and homosexual individuals. While our findings do not indicate which thalamus nuclei were more or less involved, the results point towards the idea that GMV differences in this region may change reward propensities by weighing the stimuli associated with one’s preferred sex more rewarding relative to the ones associated with one’s less desired sex. Nevertheless, our results preclude the possibility that these morphological differences between homo- and heterosexual individuals reflect atypical cerebral sex dimorphisms.

Recent studies showed that the precentral gyrus is implicated in the regulation of emotion and self-evaluation^51^, including the perception of body image^52^. Moreover, the precentral gyrus was associated with self-motion control of penile movement as well as with the imagination of sexual behaviors during sexual arousal when visual stimuli were presented^53^. Homosexual men showed decreased fractional amplitude of low-frequency fluctuation (fALFF) in the left postcentral gyrus^54^ compared to heterosexual men, whereas both heterosexual and homosexual individuals exhibited higher activity of the post-central gyrus during subjective arousal^55^. Together with the thalamus, precentral regions showed higher activation in heterosexual individuals while watching female-to-female and female-to-male sexually arousing videos. In contrast, the right hippocampus and right precentral gyrus were activated by female-to-male and male-to-male sexual stimuli^56^. Hence, these regions are likely involved in sexual arousal. Moreover, the precentral gyrus region was the region which was shared in both contrasts between HeM vs. HoM and HeW vs. HoW. This potentially indicates the role of this region in sexual behavior/sexual arousal regardless of biological sex.

The examination of the functional fingerprint of the thalamus and pre-/ and post-central gyri is consistent with existing literature in that these regions are involved in processing incoming sensory information. Analyzing the thalamus seed revealed its role in functional domains including action execution, perception, pain monitoring, and reward processing. Similarly, the pre-/ and post-central gyri seeds were functionally related to broader cognitive processes including action execution, speech, perception, and motor learning. The similarity in functional domains for both regions is not surprising since the thalamus and sensorimotor areas are functional and structurally connected by fiber projections to the somatosensory and sensorimotor cortices or so-called thalamocortical radiation^49,57,58^. These pathways from thalamic nuclei to the sensorimotor areas mediate the interaction between attention and arousal in humans^59^ as well as having a putative role in multisensory information integration^60^ and processing of nociceptive and non-nociceptive information^61^. Moreover, these two regions were part of the prediction map acquired by the automatic meta-analytical tool (NeuroQuery) based on studies associated investigating sexual orientation. Sexual orientation, therefore, has strong associations with areas primarily linked to processing and integrating incoming sensory, reward-related, and motor information, but it may also be plausible that these differences are a result of different physical activity levels (e.g. sports engagement) or are at least partially contingent on how much effort individuals invest to obtain rewards.

The putamen emerged as the only region with increased gray matter volume in homosexual versus heterosexual individuals but the difference was driven by homosexual women. Interestingly, however, the comparison between heterosexual and homosexual women further revealed morphological differences in the caudate body. One interpretation could be in the context of sexual dimorphism. Larger putamen GMVs among HoW could be a token of masculinization, as previously larger GMV was observed in men relative to women^7^. Nevertheless, our additional analysis did not yield any significant correlation results in men. Instead, we believe these regions may be more involved in women’s sexual behavior. In line with this interpretation, a study by^62^ highlighted higher activation in the striatum for the comparison of HoW > HeW, revealing a bias towards same-sex faces^63^. Moreover, the caudate body and ventral pallidum were meta-analytically identified as key regions mediating emotional and social attachment as related to the unconscious activation of bonding mechanisms during sexual stimulation in women^15^.

Early studies reported a volumetric increase of the suprachiasmatic nucleus of the hypothalamus^16^ but smaller volumes of the third interstitial nucleus of the anterior hypothalamus^17^ in HoM relative to HeM, as well as that HoM displayed thicker anterior cingulate cortex, precuneus, and left occipito-termporal cortex relative to HeM and HeW^21^. Our results, however could not confirm these early findings. Apart from not taking into account and correcting for endogenous hormonal levels across our sample, further potential reasons could entail methodological differences of these early studies. The differences between early studies’ results, taken together with the present study, further stress the necessity for carefully designed and interpreted studies on the morphologic brain differences in sexual orientation. These VBM results, however, reinforce the concept that gray matter differences in sexual orientation are rather focal instead of diffuse and equally distributed in all regions.

## Limitations

Sexual orientation is hypothesized to be related to prenatal exposure to sex hormones^64^. It is thus plausible to assume that differences in brain structure could be explained by the targeted effects of sex hormones on the thalamus, hypothalamus, and the hippocampus^65,66^. While we collected hormonal data prior to MRI measurements, we were unable to include them into the analyses as a large proportion of this data is missing due to technical limitations. Future studies should systematically assess the effects of hormonal, genetic, and epigenetics factors on structural brain differences. Moreover, we did not collect data on sexual activity and gender atypicality which might therefore introduce group differences. Although, to the best of our knowledge, there is no evidence for an association between sexual activity and brain morphological phenotypes, future studies should assess markers of sexual activity to examine potential effects of gender atypicality within homosexual and heterosexual groups. In addition, future studies should investigate the effects of gender atypicality on morphological changes longitudinally in a neurodevelopment context. Given the multidimensionality and complexity of human sexual behavior, future studies should strive to disentangle sensory perception from motivation and cognitive processes when examining the effects of sexual orientation on brain structure and function.

## Conclusion

In conclusion, we showed that sexual orientation is associated with distinct changes in brain structure and that these effects vary with biological sex. Altogether, our findings show that sexual orientation has strong associations with areas primarily linked to processing and integrating incoming sensory, reward-related, and motor information. The findings aid the understanding of the neurobiology of sexual orientation and emphasizes the need of including or controlling for potential effects of the sexual orientation of participants in neuroimaging studies. Moreover, results provide new insights into sexual behavior in general and have implications for healthcare policies.

## Supplementary Information

Supplementary Information
