# Supplementary material for: Brain structure changes associated with sexual orientation
Source: Sci Rep. 2021 Mar 3;11:5078. doi: 10.1038/s41598-021-84496-z (PMC7930173; doi:10.1038/s41598-021-84496-z)
Supplement: Supplementary file 1 — Supplementary Information [file 41598_2021_84496_MOESM1_ESM.docx]

# Brain structure changes associated with sexual orientation

*Mikhail Votinov^1,2^, Katharina S. Goerlich^3^, Andrei A. Puiu^2^, Elke Smith^4^, Thomas Nickl-Jockschat^5,6^, Birgit Derntl^7^, Ute Habel^1,2^*

**Supplementary data**

**RESULTS**

There were no significant age or educational attainment level differences between the four groups (p > .05 for all tests).

**Supplementary Table 1**. Sample demographics.

|  | Men (n=37) | | Women (n=37) | |
| --- | --- | --- | --- | --- |
|  | Heterosexual | Homosexual | Heterosexual | Homosexual |
| N per group | 16 | 21 | 18 | 19 |
| Age (means ± SD) | 32.38 ± 10.92 | 26.33 ± 5.50 | 32.28 ± 12.07 | 28.63 ± 6.08 |
| Dexterity | 14 | 21 | 17 | 17 |
| Years of education^a^ | 14.88 ± 3.14 | 16.38 ± 1.69 | 14.78 ± 3.21 | 15.37 ± 2.29 |

^a^10 years of education: O-level; 13 years of education = A-level; 16 years of education 0 bachelor’s degree; 18 years of education 0 master’s degree.

**Personality data**

**Supplementary Table 2**. Personality dimensions and accentuations.

|  | Male (n=37) | | Female (n=37) | |
| --- | --- | --- | --- | --- |
|  | Heterosexual (n=16) | Homosexual (n=21) | Heterosexual (n=18) | Homosexual (n=19) |
| BSRI Gender roles | | | | |
| Masculinity | 5.22 ± 0.14 | 4.72 ± 0.12 | 4.51 ± 0.13 | 4.73 ± 0.12 |
| Femininity | 4.76 ± 0.13 | 4.77 ± 0.12 | 4.77 ± 0.13 | 4.85 ± 0.19 |
| TAS-20 Alexithymia | | | | |
| Total Score**^^** | 38.08 ± 5.91 | **46.33 ± 9.50** | 39.62 ± 8.93 | **46.21 ± 7.26** |
| DIF | 10.85 ± 2.07 | 13.19 ± 4.37 | 13.08 ± 3.84 | 12.89 ± 5.02 |
| DDF | 10.46 ± 3.38 | 11.14 ± 2.83 | 9.02 ± 3.09 | 10.53 ± 2.06 |
| EOT**^^** | 16.77 ± 3.96 | **21.95 ± 6.02** | 17.38 ± 4.17 | **22.79 ± 4.48** |
| NEO-FFI Big Five Personality Traits | | | | |
| Neuroticism***^^** | **27.42 ± 6.24** | 32.52 ± 8.05 | **36.91 ± 8.81** | 28.13 ± 7.53 |
| Extraversion | 42.92 ± 6.95 | 39.43 ± 8.65 | 42.18 ± 6.89 | 39.94 ± 6.39 |
| Openness**^^** | **48.25 ± 4.33** | 43.62 ± 6.87 | 36.91 ± 8.81 | 41.38 ± 9.20 |
| Agreeableness | 43.08 ± 4.98 | 44.62 ± 5.20 | 46.09 ± 3.81 | 45.00 ± 9.51 |
| Conscientiousness | 46.42 ± 6.13 | 41.71 ± 6.21 | 43.73 ± 8.57 | 44.18 ± 5.76 |
| ICP Clinical Personality Accentuations | | | | |
| Paranoid | 45.39 ± 3.27 | 45.19 ± 2.57 | 46.43 ± 3.15 | 43.68 ± 2.70 |
| Dependent | 47.69 ± 2.34 | 51.43 ± 1.84 | 49.64 ± 2.25 | 48.16 ± 1.93 |
| Impulsive-explosive | 45.00 ± 2.45 | 48.57 ± 1.93 | 48.21 ± 2.36 | 43.68 ± 2.03 |
| Schizoid***** | 43.08 ± 2.45 | **50.71 ± 1.93** | 47.50 ± 2.36 | 45.00 ± 2.03 |
| Narcissistic***^^** | **50.00 ± 1.97** | **50.95 ± 1.55** | 46.82 ± 1.63 | 45.00 ± 1.90 |
| Borderline | 47.69 ± 2.48 | 48.57 ± 1.95 | 47.57 ± 2.39 | 45.53 ± 2.05 |
| Avoidant***** | 45.39 ± 2.70 | **53.33 ± 2.13** | 49.71 ± 2.60 | 43.42 ± 2.23 |
| Compulsive***** | 44.23 ± 2.77 | **51.67 ± 2.18** | 49.29 ± 2.67 | 46.32 ± 2.29 |
| Schizotypal***^^** | **48.46 ± 2.29** | **48.57 ± 1.80** | 45.36 ± 2.20 | 43.42 ± 1.89 |
| Antisocial***^^** | **48.07 ± 2.30** | **49.29 ± 1.81** | 42.14 ± 1.90 | 42.63 ± 1.90 |
| Histrionic | 46.15 ± 2.51 | 48.57 ± 1.97 | 44.29 ± 2.42 | 45.79 ± 2.08 |

BSRI = Bem Sex Role Inventory. TAS-20 = Toronto Alexithymia Scale. DIF = difficulty identifying feelings, DDF = difficulty describing feelings, EOT = externally oriented thinking. NEO-FFI = NEO Five Factor Inventory. *****main effect of biological sex; **^ ^**main effect of sexual orientation; ***^^**interaction biological sex × sexual orientation.

**Results for Mean Grey Matter (GM), White Matter (WM), and Cerebrospinal Fluid (CSF).**

**Supplementary Table 3**. Mean Grey Matter (GM), White Matter (WM), and Cerebrospinal Fluid (CSF) volumes (ml) of heterosexual and homosexual men and women.

|  | | **GM** | | **WM** | **CSF** | **n** |
| --- | --- | --- | --- | --- | --- | --- |
| **Men** | | 783.1±61.9 | | 574.54±47.3 | 347.11±54.5 | 37 |
| **heterosexual** | | | 779.9±70.4 | 562.5±45.3 | 334.04±41.8 | 16 |
| **homosexual** | | | 785.51±56.3 | 583.8±47.7 | 357.1±61.6 | 21 |
| **Women** | | | 713.22±59.9 | 513.2±46.2 | 309.5±41.4 | 37 |
| **heterosexual** | | | 704.72±52.0 | 497.8±51.9 | 309.5±39.1 | 18 |
| **homosexual** | | | 721.3±57.7 | 527.8±35.3 | 309.5±44.5 | 19 |
| **Total** | **heterosexual** | | 740.1±71.4 | 528.2±58.3 | 321.1±41.7 | 34 |
| **homosexual** | | | 755.0±64.9 | 557.2±50.5 | 334.5±58.6 | 40 |
| **Total** | | | 748.1±67,9 | 543.9±55.7 | 328.3±51.6 | 74 |

**Overlay with model derived for NeuroQuery**

These findings support our initial predictions and suggest that morphological differences associated with sexual orientation are linked to neurobehavioral phenotypes.

Supplementary Figure 1. Overlap between VBM whole-analyses and results from NeuroQuery.


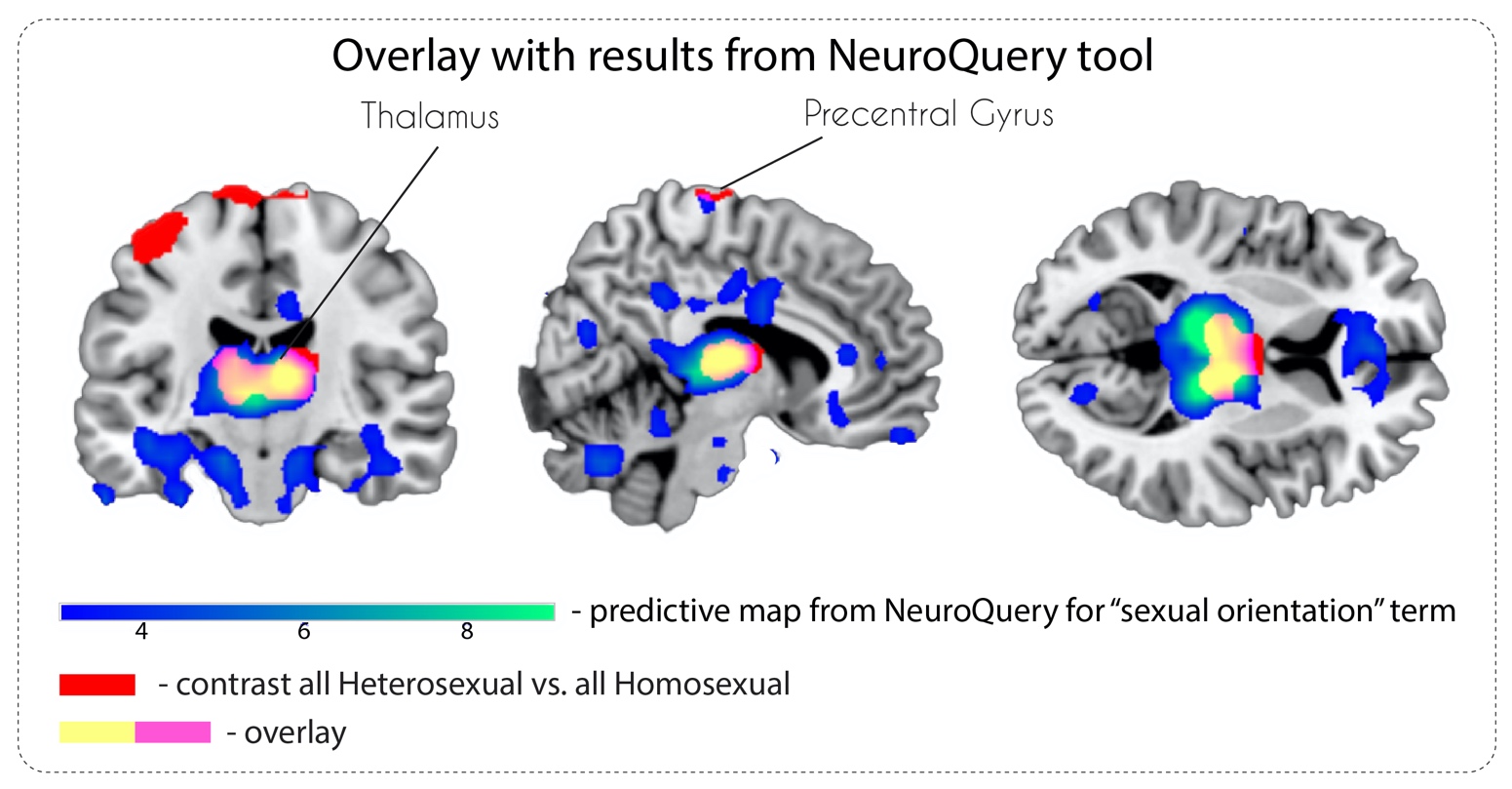


**ROI analyses - Relationship between extracted GMV from clusters and gender roles self-concepts**

Partial correlations between the ensuing GMV of the main analyses above, biological sex and sexual-orientation revealed an association between a more feminine self-concept and more GMV in the thalamus for HeW participants only (partial r = .57, *p* = 0.02), see Supplementary Figure 2.

Supplementary Figure 2. HeM = heterosexual men; HoM = homosexual men; HeW = heterosexual women; HoW = homosexual women.


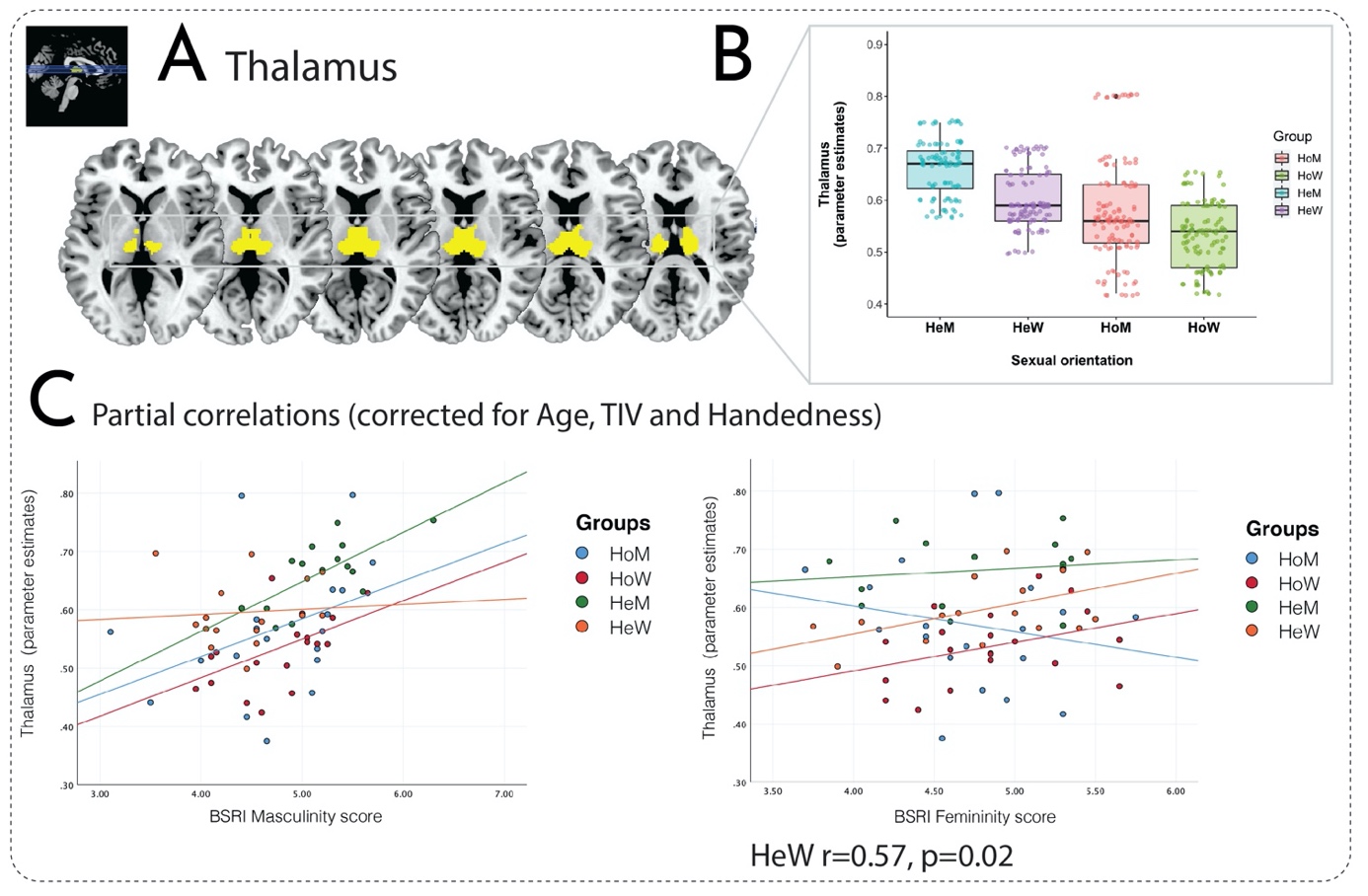


Partial correlations between femininity scores and GMV of the precentral gyrus revealed a positive correlation for HeW r =.59, *p* =.017 and partial correlations between masculinity scores and GMV of the precentral gyrus a positive correlation with for HoM r = .40, *p* =.048, only. See Supplementary Figure 3.

*Supplementary Figure 3*. *HeM = heterosexual men; HoM = homosexual men; HeW = heterosexual women; HoW = homosexual women.*


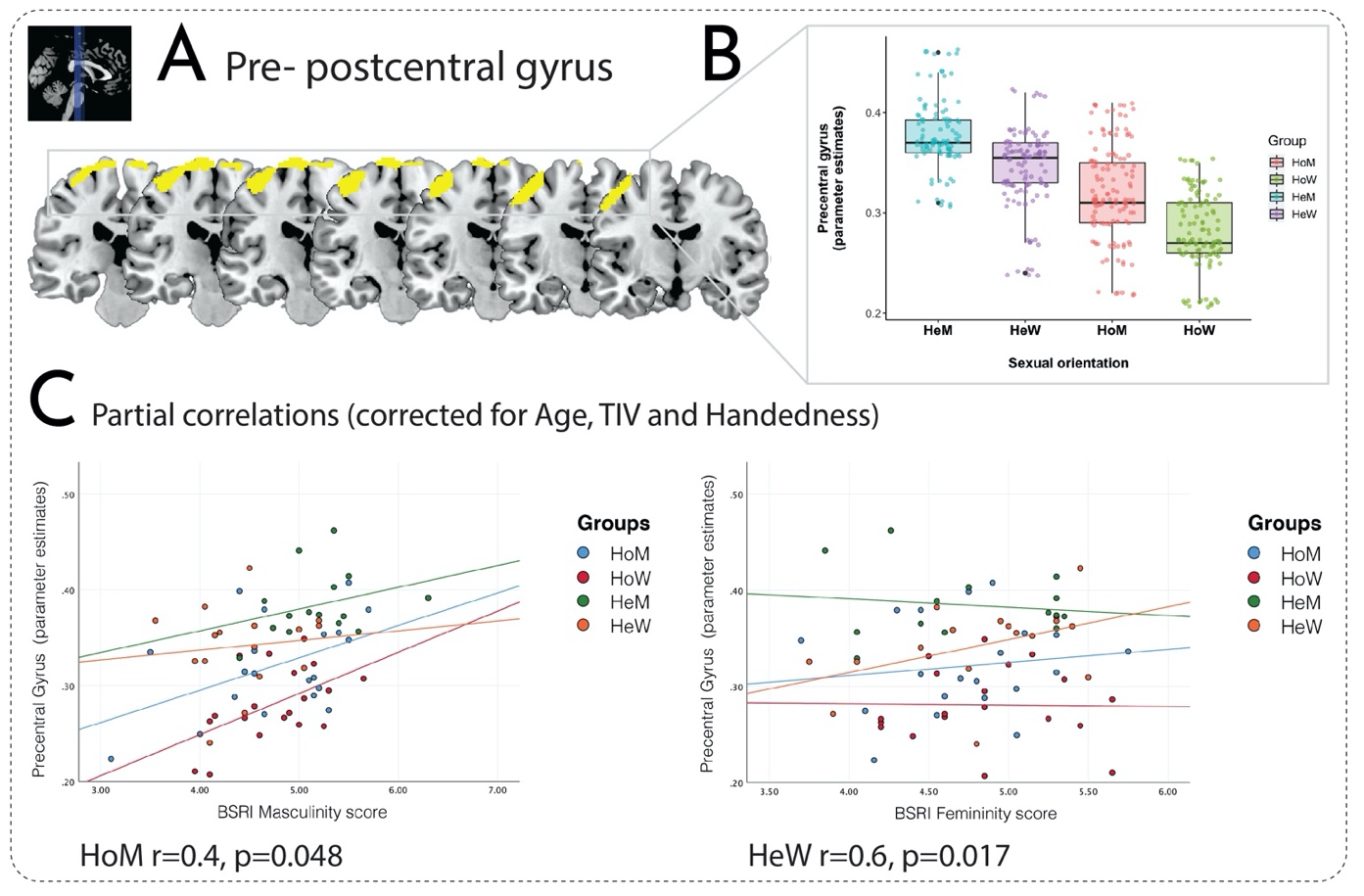


Correlation between GMV of the putamen and femininity scores revealed a negative correlation for HeM r = -.56, *p* =.029 and a positive correlation for HoM r = .51, *p* =.016. See Supplementary Figure 4.

*Supplementary Figure 4. HeM = heterosexual men; HoM = homosexual men; HeW = heterosexual women; HoW = homosexual women.*


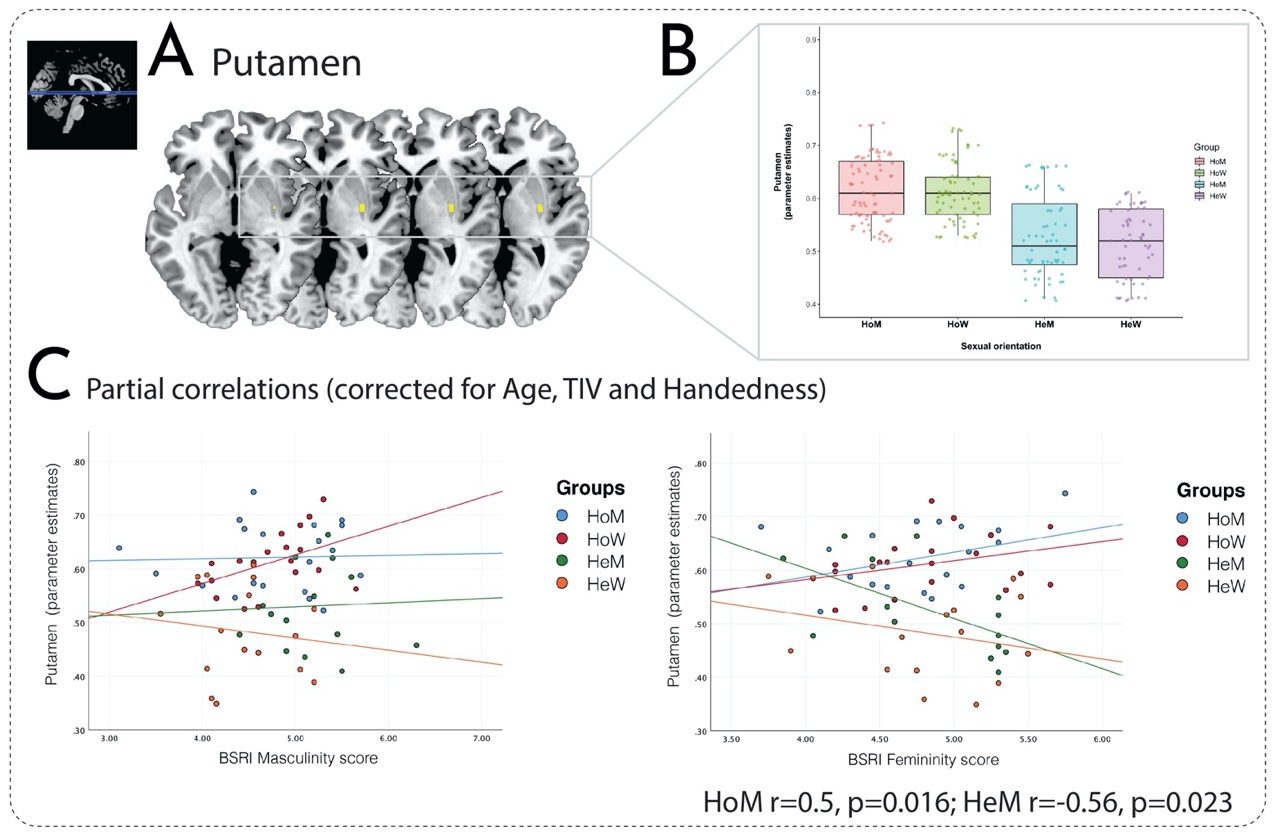


**Contrast HeM > HeW and HeW > HeM**

The contrast HeM>HeW revealed difference in right amygdala (threshold p < 0.05 FWE corrected). While results for reverse contrast did not survive after p < 0.05 FWE correction. The results are listed in the table below.

**Supplementary Table 4**. Cluster list for contrast HeM > HeW (threshold p < 0.05 FWE corrected at voxel level).

| **Brain areas (aal)** | **Side** | **Cluster size** | **x** | **y** | **z** | **T** |
| --- | --- | --- | --- | --- | --- | --- |
| Amygdala | R | 32 | 24 | -9 | -30 | 5,50 |
|  |  |  |  |  |  |  |

*Note:* L/R = left/right in the brain;
